# Core modular blood and brain biomarkers in social defeat mouse model for post traumatic stress disorder

**DOI:** 10.1186/1752-0509-7-80

**Published:** 2013-08-20

**Authors:** Ruoting Yang, Bernie J Daigle Jr, Seid Y Muhie, Rasha Hammamieh, Marti Jett, Linda Petzold, Francis J Doyle

**Affiliations:** 1 Institute for Collaborative Biotechnologies, University of California Santa Barbara, Santa Barbara, CA 93106-5080, USA; 2 Department of Computer Science, University of California Santa Barbara, Santa Barbara, CA 93106-5080, USA; 3 Department of Mechanical Engineering, University of California Santa Barbara, Santa Barbara, CA 93106-5080, USA; 4 Department of Chemical Engineering, University of California Santa Barbara, Santa Barbara, CA 93106-5080, USA; 5 US Army Center for Environmental Health Research, Fort Detrick, MD, 21702-5010, USA; 6 Advanced Biomedical Computing Center, Frederick National Laboratory for Cancer Research, Frederick, MD 21702-5010, USA

## Abstract

**Background:**

Post-traumatic stress disorder (PTSD) is a severe anxiety disorder that affects a substantial portion of combat veterans and poses serious consequences to long-term health. Consequently, the identification of diagnostic and prognostic blood biomarkers for PTSD is of great interest. Previously, we assessed genome-wide gene expression of seven brain regions and whole blood in a social defeat mouse model subjected to various stress conditions.

**Results:**

To extract biological insights from these data, we have applied a new computational framework for identifying gene modules that are activated in common across blood and various brain regions. Our results, in the form of modular gene networks that highlight spatial and temporal biological functions, provide a systems-level molecular description of response to social stress. Specifically, the common modules discovered between the brain and blood emphasizes molecular transporters in the blood-brain barrier, and the associated genes have significant overlaps with known blood signatures for PTSD, major depression, and bipolar disease. Similarly, the common modules specific to the brain highlight the components of the social defeat stress response (e.g., fear conditioning pathways) in each brain sub-region.

**Conclusions:**

Many of the brain-specific genes discovered are consistent with previous independent studies of PTSD or other mental illnesses. The results from this study further our understanding of the mechanism of stress response and contribute to a growing list of diagnostic biomarkers for PTSD.

## Background

Post-traumatic stress disorder (PTSD) is an anxiety disorder that is triggered after exposure to traumatic events. Individuals with PTSD have persistent fear memory and often feel emotionally numb. If left untreated, PTSD can be life-threatening, as it is often linked with substance abuse and severe depression. A study of 289,328 Iraq and Afghanistan veterans who were first-time users of Veterans Affairs (VA) health care between 2002 and 2008 showed that 22% of veterans were diagnosed with PTSD and 17% were diagnosed with depression [1]. Given the predominance of PTSD and its negative consequences to long term health, it is very important to identify measurable and quantifiable biological parameters, i.e., biomarkers, which can serve as prognostic and diagnostic indicators for PTSD. Recent studies have proposed several candidate brain gene biomarkers that are associated with PTSD [2,3]. Even though PTSD is an illness of the brain, taking brain biopsy or spinal fluid is not a viable option for diagnosis. Instead, blood can be used as a surrogate for brain tissue for the purpose of identifying biomarkers [4-8]. Specifically, Rollins et al. recently found over 4,100 brain transcripts co-expressed in the blood of healthy human subjects [9]. Furthermore, it was shown that the mRNA levels of certain transcripts in PTSD patients remain changed with respect to controls even 16 years after the traumatic event [8,10]. Thus, blood gene expression assays are of particular interest for both short-term and long-term diagnosis, prognosis, and treatment of PTSD. However, the identification of predictive blood markers requires the accurate separation of biologically relevant core markers from unrelated downstream signals. This task is particularly challenging when using surrogate tissues, since biological noise from the surrogate is confounded with noise from the primary tissue. Fortunately, studies performed with model organisms allow the direct assay of both surrogate and primary tissues. By characterizing the molecular changes present in both tissues simultaneously, we can more effectively filter out spurious signals in the surrogate.

We recently used repeated exposures of mice to a trained aggressor mouse as a “social defeat” model for evaluating PTSD symptoms [11]. This social defeat model has often been used to induce anxiety, depression-like and avoidance symptoms, which are the most prominent psychiatric features of PTSD and common co-morbidities. Using a “cage-within-cage resident-intruder” protocol (designed to model unpredictable threats of daily trauma), we exposed individual subject male C57BL/6 J mice to single aggressors for six hours daily for 5 or 10 days, and we placed individual control subject mice in the same cages but in the absence of any other mice. After allowing the subject animals to recover for either 1 or 10 days (5 day exposure) or 1 or 42 days (10 day exposure), we then collected tissue samples of blood and seven brain regions of mice under the different stress conditions and measured gene expression levels of these tissues using DNA microarrays. As described in [11], the durations of aggressor exposure were chosen to simulate shorter term (5-day) and longer term (10-day) stress. The shortest recovery phase duration (1 day) was chosen to study the immediate effects of stress. The longer of the two recovery phase durations for each exposure time were selected based on behavioral tests conducted throughout the study. These tests demonstrated 5-day exposure defeated mice showed signs of recovery around 10 days post-exposure, and 10-day exposure defeated mice showed signs of recovery at much longer times (up to 42 days post-exposure). Because PTSD represents a persistent stress response, it is important to identify differentially expressed genes (DEGs) active both immediately after the exposure and after a long recovery period. Thus, in the current work we focus on genes consistently over-/under-expressed across all experimental conditions, rather than on DEGs from individual conditions (we will address the latter in future work). The seven brain regions analyzed in this study were chosen due to their known roles in fear memory formation, emotion regulation, and decision-making—all processes important to the development and pathology of PTSD [3]. In particular, the amygdala regulates fear memory and emotional aspects; the hippocampus is the center for short term memory, and the prefrontal cortex controls decision-making. In addition, the ventral striatum is strongly associated with emotional and motivational aspects of behavior, the stria terminalis serves as a major output pathway of the amygdala, and the septal area plays a role in reward and reinforcement along with the ventral striatum. We note that a similar protocol has also recently been used to profile social defeat-induced gene expression changes in the nucleus accumbens, ventral tegmental area, and blood plasma [12-14].

The field of systems biology has demonstrated that complex diseases such as PTSD are not caused by changes in a single gene or pathway. Rather, changes occur in a hierarchy of gene modules which collectively contribute to disruption of essential cellular functions [15-17]. To characterize this module hierarchy, many researchers have adopted an unsupervised approach [17-25] that constructs a network based on gene expression data and identifies functional modules based on network topology or “guilt-by-association”. However, these methods usually face the problem of under-determination, where the number of interactions to be inferred far exceeds the number of independent measurements [22]. Other studies have adopted a supervised network identification approach that begins with a list of “seed” genes and gradually expands the list by adding interacting genes, ultimately resulting in a compact gene module network [26-28]. These supervised approaches have shown good performance for classification tasks, and we expand upon one of them in this work.

Previous computational and experimental work suggests that functional gene modules are highly conserved across conditions, tissues, and species [17,29,30]. Direct comparisons have been made between multiple mouse tissues [21], between human and mouse brains, and between human blood and brain tissue [4]. However, modules inferred separately from different conditions yield partial overlaps at best, which makes drawing comprehensive biological conclusions difficult. Recently, we developed a new module identification tool entitled COMBINER (COre Module Biomarker Identification with Network Exploration) that identifies distinct conserved expression modules across various conditions. The fundamental idea behind COMBINER is to infer candidate modules from data of one condition and validate the inferred modules in other conditions using supervised classification. Those candidate modules that perform well in classifying samples from multiple conditions are then defined as “core modules”. There are three advantages to this approach: (1) The resulting modules are compact and thus exclude unrelated downstream signals; (2) The modules are distinct and well-defined with respect to which conditions/tissues/species invoke them; (3) This method provides multiple robust discriminative biomarkers co-validated in at least two experimental conditions. Given these advantages, we have applied a customized version of COMBINER to mouse social defeat gene expression data deriving from seven brain regions along with blood to identify common expression modules.

In this work, we have attempted to answer two biological questions: *1. Which expression modules act in common between blood and brain tissue of the social defeat mouse model? 2. Which modules act in common between different brain regions?* To do so, we first performed a pair wise comparison of differential gene expression, biological pathways, and GO terms between tissues. We then applied a new version of COMBINER which we modified in two ways (discussed below). First, we used linear models to deconvolve time-dependent effects on gene expression from effects due to social defeat. Second, we developed an improved consensus feature elimination method to identify robust modules from data with a relatively small sample size. Our results, in the form of blood-brain and brain-brain social defeat core module networks, provide a concise biological description of social defeat and generate many candidate PTSD biomarkers for future study.

## Results and discussion

### Overlaps of DEGs/DEGOs/DEPaths

**Figure 1:**
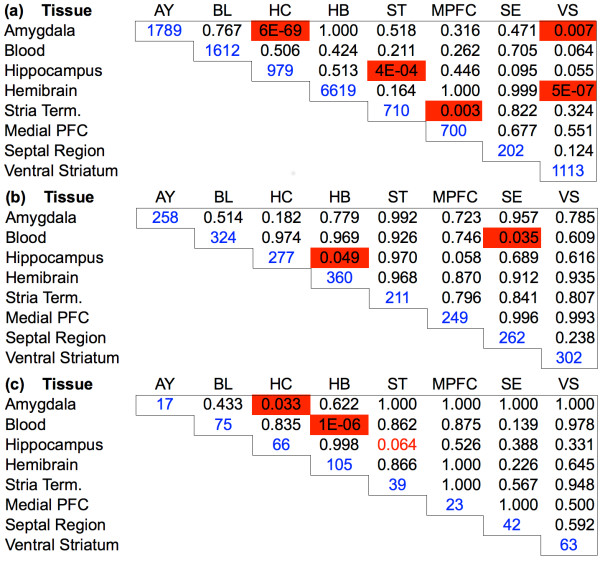
**Overlap analysis between blood and brain regions.** Numbers of significant DEGs **(a)**, DEGOs **(b)**, and DEPATHs **(c)** are listed in blue on the diagonal, while hyper geometric p-values are listed in the off-diagonal. P-values ≤ 0.05 are considered significant (cells highlighted in red). We consider the DEPATH overlap between hippocampus and stria terminalis to be marginally significant (red font), as it has a p-value ≤ 0.1 and is supported by a highly significant DEG overlap between the same tissues. (HB: hemibrain (hemisphere), AY: amygdala, HS: hippocampus, MPFC: medial prefrontal cortex, VS: ventral striatum, SE: septal region and ST: stria terminalis).

First, we identified differentially expressed genes (DEGs) in each individual tissue across all time points using a limma moderated t-test [31]. The numbers of significant DEGs (p ≤ 0.05) for each tissue are listed in blue on the diagonal in Figure 1a. We then established the significance of DEG overlaps by computing a hypergeometric p-value for each pairwise tissue combination (listed in the off-diagonal). Hypergeometric p-values ≤ 0.05 are considered significant (cells highlighted in red in Figure 1a).

Next, we identified differentially expressed Biological Process GO terms (DEGOs) in each tissue by first ranking all genes in descending order of limma significance and then performing Iterative Group Analysis (iGA) [32] for each GO term with ≤ 100 constituent genes. We computed p-values for each term’s iGA score using a null distribution obtained via 1000 random permutations of the original gene order. The numbers of significant DEGOs (p ≤ 0.05) for each tissue are listed in blue on the diagonal in Figure 1b. We established the significance of DEGO overlaps in the same manner as in Figure 1a.

Finally, we identified differentially expressed MSigDB [33] (http://www.broadinstitute.org/gsea/msigdb/) canonical sub-pathways (DEPATHs) in the same manner as DEGOs with the following modification. For each pathway, we performed iGA separately for all ordered sub-pathways ranging in size from three to 10 genes (when genes are ordered in terms of limma significance). We selected the highest scoring sub-pathway and established significance as before by repeating the procedure on 1000 random gene order permutations. The numbers of significant DEPATHs and significant DEPATH overlaps are denoted in the same manner as above.

The overlaps of particular interest include amygdala-hippocampus (AY-HC) and hippocampus-stria terminalis (HC-ST), as these two scored significantly in the DEG comparison and significantly or nearly significantly, respectively, in the DEPATH comparison. These DEPATHs describe processes such as inflammation, diabetes, apoptosis, and immune response. Tables 1 and 2 show the significantly overlapping DEPATHs of AY-HC and HC-ST, respectively. We list the original name of each sub-pathway along with the following information from the iGA sub-pathway analysis conducted on the hippocampus data: number of genes in the highest scoring sub-pathway (Sig. Genes), sub-pathway permutation p-value, and Benjamini-Hochberg corrected sub-pathway false discovery rate (FDR). We note that none of these pathways would have been identified as significant from the hippocampus data alone when using a FDR ≤ 0.05 cut-off. We also note significant overlaps in the blood-septal region and blood-Hemibrain comparisons, where DEGOs related to apoptosis and DEPATHs related to insulin/diabetes, respectively, were identified. Additional file 1: Table S1 and Additional file 2: Table S2 contains detailed lists of these DEGOs and DEPATHs, respectively.

**Table 1:** Significantly overlapping DEPATHs between amygdala and hippocampus

**Name**	**Number of significant genes**	**p-value**	**FDR**
BioCarta cytokine pathway	9	<0.002	<0.220
BioCarta inflam pathway	8	0.002	0.220
KEGG type I diabetes mellitus	7	0.012	0.440
KEGG JAK STAT signaling pathway	10	0.014	0.456

FDR denotes false discovery rate.

**Table 2:** Significantly overlapping DEPATHs between hippocampus and stria terminalis

**Name**	**Number of significant genes**	**p-value**	**FDR**
SA caspase cascade	6	0.005	0.400
BioCarta IL1R pathway	10	0.010	0.440
KEGG cytosolic DNA sensing pathway	9	0.021	0.499
KEGG graft versus host disease	6	0.029	0.562
KEGG prostate cancer	9	0.030	0.562
BioCarta keratinocyte pathway	9	0.046	0.636

### Core module network

**Figure 2:**
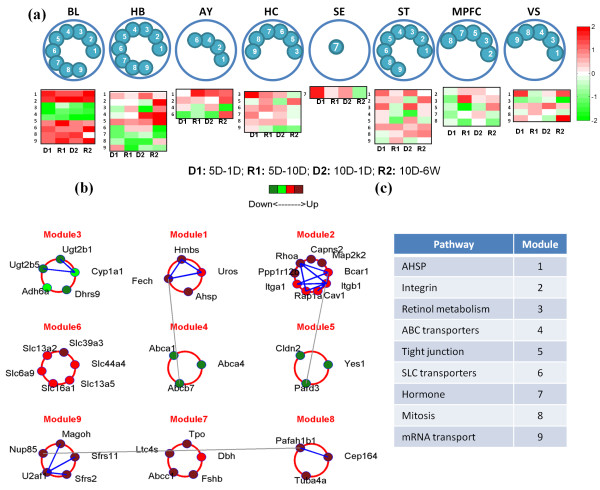
**Blood-brain network. (a)** nine expression modules resulted from consensus feature elimination; their brain-specific expression locations are indicated with numbered blue circles. Time-specific blood expression patterns of each module are displayed using average time curves in the form of expression panels. **(b)** the blood expression level of each gene in the nine modules is indicated with a colored circle. Known protein-protein interactions (PPIs) are marked by lines connecting genes—blue lines denote within-module interactions, while gray lines denote between-module interactions. **(c)** the putative biological functions of the expression modules are listed (as inferred using the KEGG annotation). (HB: hemibrain (hemisphere), AY: amygdala, HS: hippocampus, MPFC: medial prefrontal cortex, VS: ventral striatum, SE: septal region and ST: stria terminalis; 5D-1D/10D: 5 day treatment, 1 day/10 day recovery, 10D-1D/ 6 W: 10 day treatment, 1 day/6 week recovery).

**Figure 3:**
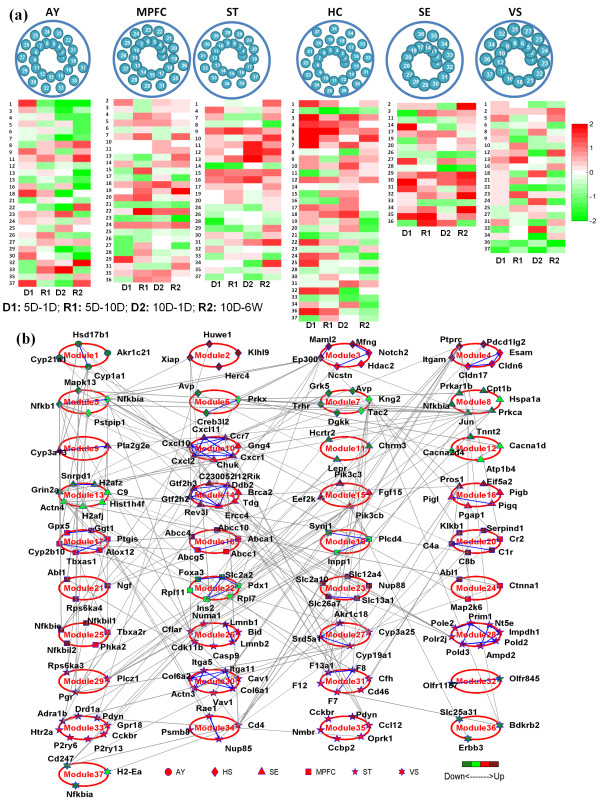
**Brain-brain network. (a)** application of COMBINER to brain data yields thirty-seven core modules. The tissue- and time-specific expression patterns of each module are presented in the same manner as before. **(b)** the expression levels and known PPIs of the core module genes are displayed. The shape of a gene represents its inference region, and the color denotes its expression level in that region. Blue lines denote known within-module protein-protein interactions (PPIs), while gray lines denote between-module PPIs. (HB: hemibrain (hemisphere), AY: amygdala, HS: hippocampus, MPFC: medial prefrontal cortex, VS: ventral striatum, SE: septal region and ST: stria terminalis; 5D-1D/10D: 5 day treatment, 1 day/10 day recovery, 10D-1D/ 6 W: 10 day treatment, 1 day/6 week recovery).

Although the differential expression overlap analysis provided some biological insight into the pairwise molecular similarities between mouse tissues during social defeat, overlap results between DEGs, DEGOs, and DEPATHs were not always consistent. Overlap analysis between multiple tissues is more desired, while these overlaps are very limited due to the high noise-to-signal ratio of microarray. In addition, it was not obvious how best to combine the results into an overall biological description of mouse social defeat. Thus, we turned to a network-level analysis to provide deeper insight. Because the desired diagnostic biomarkers should be generally over-expressed in both the stress treatment and recovery period, we extended the COMBINER method [28] to accommodate all four conditions, which resulted in multiple-time-segment data. However, we would expect an age effect in the control mice. For example, the gene expression patterns of control mice in the 10-day treatment 1-day recovery and 10-day treatment 42-day recovery groups were significantly different due to mouse age. Thus, we used the limma software [31] to deconvolve the undesired effects of differing mouse ages as explanatory variables in a linear model, and we subtracted these variables from the original gene expression values. We then applied COMBINER to the “time standardized” data to construct a blood-brain network (common modules co-expressed in blood and seven brain regions, Figure 2) and a brain-brain network (common modules co-expressed in six brain regions, Figure 3).

### Blood-brain network

We first investigated the expression modules active in both blood and multiple brain regions. Starting with the top 100 candidate modules (when ranked by pathway activity absolute t-score—see Methods) inferred from blood sample data, we identified modules that were also active in each brain region. To do so, we removed features using Consensus Feature Elimination until the average classification Area Under the ROC Curve (AUC) evaluated on each brain region exceeded 0.75 (see [28] for additional details). After repeating this procedure separately for all brain regions, a total of nine core modules remained. Figure 2a presents each module’s brain region-specific expression patterns. We used average time curves (see Methods) to show the time-specific expression pattern of the modules as heat maps in Figure 2a. Figure 2b further shows the expression of the core modules and the protein-protein interactions (PPIs) between their gene products. The color of each gene denotes its expression level in the blood. Blue lines denote known PPIs within modules, while gray lines denote known PPIs between modules. Figure 2c lists the putative biological functions of the core modules; detailed module information is summarized in Additional file 3: Table S3. We note that use of COMBINER resulted in seven discriminative blood biomarker sets (average 0.81 mean AUC and 0.26 mean error rate) which have each been validated using data from one of the brain regions. Table 3 lists the final number of modules identified from each blood-brain region pair with the associated mean AUC and mean error rate.

**Table 3:** Identified final modules between blood and brain regions with the associated mean AUC and mean error rate of both tissues

**Validation**	**HB**	**AY**	**HC**	**ST**	**MPFC**	**SE**	**VS**	**Core module**	**Core gene**
**Inference**									
Blood	92	20	23	27	22	2	19	9	43
Final gene	171	52	51	70	72	7	44		
Mean AUC	0.73	0.85	0.79	0.86	0.76	0.82	0.84		
Mean error rate	0.24	0.24	0.29	0.22	0.31	0.27	0.24		

The mean AUC and error rate were calculated by 500 LDA classifiers with random sampling on both tissues. (*HB* hemibrain (hemisphere), *AY* amygdala, *HS* hippocampus, *MPFC* medial prefrontal cortex, *VS* ventral striatum, *SE* septal region and *ST* stria terminalis).

The resulting nine core modules represent biological functions related to molecular transport, integrin and tight junction function, retinol metabolism, cell cycle, and mRNA transcription. Although initially inferred from blood tissue, most of these processes have been previously implicated in normal and pathological brain function. For example, tight junctions and ABC efflux transporters are present in the blood-brain barrier (BBB) and the blood-cerebrospinal fluid barrier (BCSFB) [34,35], and SLC transporters encode facilitated transporters and ion-coupled secondary active transporters such as neurotransmitters. The latter also represent the major class of transporters used in the delivery of drugs to the brain [36]. In addition, overexpressed integrin genes lead to vascular remodeling, which is believed to be highly correlated with mild Traumatic Brain Injury (mTBI) [37], a disease related to PTSD. Finally, retinoids are important for the maintenance of the nervous system and may play a role in Alzheimer’s disease [38].

The resulting 43 core genes also exhibit ample evidence for association with brain function and/or PTSD. In particular, the genes *Abca4, Fech, Magoh, Ppp1r12b*, and *Uros* were previously shown to be differentially expressed in a human PTSD signature discovered by Segman et al. [8]. Seven of the 43 genes closely resemble genes from a blood signature for depression (*Ahsp, Dhrs9, Map2k2, Slc13a2, Slc16a1, Slc39a3, U2af1*) [39,40], while *Hmbs, Pafah1b1, Sfrs2*, and *Yes1* were previously identified as bipolar disorder blood markers [41]. In addition, *Ugt2b5* and *Slc6a9* are also present in a blood signature for brain injury [42], while *Dbh, Itgb1, Ltc4s*, and *Rhoa* were reported to be relevant to mTBI [43]. Many of the other genes have been associated with various mental illnesses and neurodege-nerative diseases, including Schizophrenia, Alzheimer’s disease, and sleep disorder. Detailed associations and references are listed in Additional file 4: Table S5.

### Brain-brain network

In a similar manner as before, we first used COMBINER to infer the top 100 candidate modules for each brain region. We then identified common modules for each remaining brain region separately, removing features using Consensus Feature Elimination until the average AUC of the second region exceeded 0.75. Table 4 lists the final number of modules identified from each brain region pair, as well as the number of “core” modules and “core” genes for each brain tissue (i.e. those present in the majority of pair wise comparisons). In total, 37 core modules with 177 genes were identified in the brain-brain network.

**Table 4:** Numbers of COMBINER modules identified using data from six brain regions

**Val**	**AY**	**HC**	**ST**	**MPFC**	**SE**	**VS**	**Core module**	**Core gene**
**Inf**								
AY	/	17	25	23	0	16	1	4
HC	17	/	7	25	7	18	6	28
ST	22	29	/	23	7	22	9	45
MPFC	22	31	22	/	5	22	9	41
SE	18	26	25	17	/	28	10	53
VS	22	30	13	5	5	/	2	6

(*HB* Hemibrain (Hemisphere), *AY* amygdala, *HS* hippocampus, *MPFC* medial prefrontal cortex, *VS* ventral striatum, *SE* septal region and *ST* stria terminalis).

We list the final number of modules identified from each brain region pair, as well as the overall numbers of core modules and core genes for each region. Figure 3a displays the tissue- and time-specific expression patterns of each brain-brain core module. Figure 3b shows the expression levels of the genes in each module, as well as the known PPIs occurring between genes. Unlike the blood-brain network, the shape of a gene represents the brain region in which it was inferred. Table 5 provides the putative biological functions of the core modules as inferred, while detailed module information is summarized in Additional file 5: Table S4.

**Table 5:** The putative biological functions of the core modules in brain-brain network

**Pathway**	**Module**	**Pathway**	**Module**
Steroid	1	Complement and coagulation	20
Proteolysis	2	Neurotrophin signaling	21
Notch signaling	3	Regulation of beta cells	22
Cell adhesion	4	Transmembrane transport	23
NOD like receptor	5	Myogenesis	24
Vasopressin	6	G alpha 13 protein	25
G alpha Q protein	7	HIVNEF pathway	26
PRARA	8	Steroid	27
Linoleic acid	9	Purine	28
Chemokine signaling	10	Oocyte meiosis	29
Neuroactive ligand receptor	11	Focal adhesion	30
Muscle contraction	12	Complement and Coagulation	31
Systemic lupus erythematosus	13	Olfactory transductoino	32
DNA Repair	14	Class A1 rhodopsin like receptors	33
IRS Related events	15	Host interaction of HIV factors	34
Post translational protein modification	16	Peptide ligand biding receptors	35
Arachidonic acid	17	Calcium signaling	36
ABC Transporters	18	Downstream TCR signaling	37
Phosphadylinositol signaling	19		

In the brain-brain core module network, Modules 6, 8, 33, and 15 are of particular interest. An active Module 6 (*Creb3l2, Prkx, Avp*) in the hippocampus indicates a down regulated PKA-CREB long term potentiation pathway, which has been shown to impair memory [44]. In addition, the activity of Module 8 (*Prka1b, Hspa1a, Nfkbia, Jun, Cpt1b*) in the septal region shows down regulation of a heat shock protein (HSPA1A). Such activity has previously been found in other PTSD studies [45]. Module 33 depicts an up regulated dopamine pathway in the ventral striatum. This activity could potentially send excessive dopamine to the amygdala and other brain regions, which has been shown to lead to increased anxiety [46,47]. Finally, Module 15 implies an active pro-inflammatory response in the medial prefrontal cortex (MPFC) that agrees with the study in [48]. Other validated findings include olfactory impairment in the stria terminalis (ST) (module 32) [49]; alteration of complement pathways in the MPFC (module 20) [50] and activated coagulation function in the ST (module 31) [51].

The above findings highlight that while the putative biological functions of the brain-brain core modules largely encompass the DEPATHs identified in the statistical overlap analysis (Tables 1 and 2), the COMBINER network-based analysis provides a much richer molecular description of mouse responses to social defeat. With additional validation in human studies, we expect these findings to yield robust prognostic and diagnostic biomarkers for PTSD.

## Conclusions

The identification of diagnostic and prognostic blood biomarkers for PTSD currently is of great interest. In this work, we have improved the COMBINER method—a computational framework for identifying gene expression modules that are activated in common across experimental conditions—and applied it to blood and brain data from a mouse social defeat model. The resulting gene networks highlight stress-related biological processes active in both brain and blood and provide a comprehensive molecular description of social defeat. In total, our approach identified seven blood biomarker sets that have each been validated for classification performance in one brain sub-region. Some of the genes and processes discovered are consistent with previous independent studies of PTSD or other mental illnesses, while others represent novel candidate PTSD biomarkers. We note that the same approach can be readily applied to other disease models to construct gene networks that are activated in common across tissues; future work will focus on this task.

## Methods

### Blood, organ and tissue collection

Terminal organs, brain regions, and blood samples from subject and control C57BL/6 mice were collected after 24 hours, or 6 weeks (42 days) post 10-day social stress, and 24 hours or 1.5 weeks post 5-day social stress. Brains of C57BL/6 mice were carefully removed from the skulls, and left or right hemi-brain from each defeated or control mouse was dissected into different anatomical and functional regions: Hemibrain (Hemisphere) (HB), amygdala (AY), hippocampus (HS), medial prefrontal cortex (MPFC), ventral striatum (VS), septal region (SE) and stria terminalis (ST). The number of defeated and control mice in different regions and conditions are summarized in Table 6.

**Table 6:** Defeated and control mice (in the form of (number of defeated) / (number of control)) in different regions and conditions

**Condition**	**5D-1D**	**5D-10D**	**10D-1D**	**10D42D**
**Tissue**				
Blood	(5) / (5)	(5) / (5)	(5) / (4)	(5) / (5)
HB (Hemibrain)	5) / (5)	(5) / (5)	(5) / (5)	(5) / (5)
AY (Amygdala)	(2) / (3)	(4) / (5)	(4) / (3)	(4) / (3)
HC (Hippocampus)	(4) / (3)	(6) / (4)	(5) / (6)	(5) / (5)
MPFC (Medial Prefrontal Cortex)	(5) / (4)	(5) / (5)	(4) / (3)	(4) / (4)
SE (Septal Region)	(2) / (3)	(3) / (2)	(3) / (3)	(3) / (3)
ST (Stria Terminalis)	(5) / (5)	(5) / (5)	(4) / (4)	(5) / (4)
VS (Ventral Striatum)	(5) / (5)	(5) / (5)	(4) / (4)	(4) / (5)

(5D-1D/10D: 5 day treatment, 1 day/10 day recovery, 10D - 1D/ 6 W: 10 day treatment, 1 day/6 week recovery).

### RNA isolation and quality assessment

Total RNA was isolated according to the Trizol® method (Invitrogen Inc., Grand Island, NY) from homogenized whole blood and brain regions. RNA from blood was isolated using the PreAnalytiX PAXgene® blood RNA kit (Qiagen Inc., Valencia, CA). We collected the seven organ tissues from 5-6 control and defeat mice, respectively. We evaluated RNA integrity using the Agilent Bioanalyzer and excluded samples of low quality, which appears to either have low total RNA, or low ribosomal RNA (rRNA) mass ratio between 28S and 18S rRNA and high amount of non-ribosomal RNAs in the electropherograms.

### Microarray hybridization

Microarray assays were performed using Agilent’s genome wide mouse expression array (GE 4x44K v2 two color microarray) slides and kits (Agilent Technologies Inc., Santa Clara, CA) following the manufacturer’s protocol. To minimize batch effects, each sample was hybridized with a universal common reference that was used for all experiments. Hybridized microarray slides were scanned using Agilent Technologies Scanner G2505C US09493743.

### Microarray data processing

Genespring with feature extraction 10.x (Agilent, CA) was used to process all two-color chips. Log2 transformation, Lowess normalization, and quantile normalization were applied to normalize within and between microarrays. For the latter, we applied quantile normalization separately on data from each tissue. Outlier spots were converted to missing values. If more than half of the expression values of a probe were missing, we removed the probe from consideration. We then imputed missing values using the k-nearest neighbor imputation method. To avoid incurring a bias in favor of genes represented by a greater number of probes, we aggregated multiple probes from the same Entrez Gene together by computing the mean of the “sibling” probes. We have deposited all microarray data for this study at the Gene Expression Omnibus (GEO):

http://www.ncbi.nlm.nih.gov/geo/query/acc.cgi?acc=GSE45035.

### Linear model

We used a linear model-based approach to deconvolve the experimental time effects from the social defeat expression data. Assuming log-additive effects, our method estimates the contributions of each of the four experimental time points and subtracts them away from the remaining effect of social defeat. The linear model we used to deconvolve the experimental time effect is defined as follows:

(1) $$\left[\begin{array}{c} D_{1}\\ C_{1}\\ D_{2}\\ C_{2}\\ D_{3}\\ C_{3}\\ D_{4}\\ C_{4} \end{array}\right]=\left[\begin{array}{ccccc} 1 & 1 & 0 & 0 & 0\\ 0 & 1 & 0 & 0 & 0\\ 1 & 0 & 1 & 0 & 0\\ 0 & 0 & 1 & 0 & 0\\ 1 & 0 & 0 & 1 & 0\\ 0 & 0 & 0 & 1 & 0\\ 1 & 0 & 0 & 0 & 1\\ 0 & 0 & 0 & 0 & 1 \end{array}\right]\;\left[\begin{array}{c} \alpha_{\mathrm{defeat}}\\ \beta_{1}\\ \beta_{2}\\ \beta_{3}\\ \beta_{4} \end{array}\right]$$

where *D*_*i*_ and *C*_*i*_*i* = 1, …, 4 denote log_2_ gene expression values of defeated and control mice in condition *i*, *α*_*defeat*_ denotes the overall effect of social defeat and *β*_1_, …, *β*_4_ are the undesired time effects. In practice, we solve the above over determined system for each gene separately using least squares (implemented in the limma package), carrying forward only the gene-specific defeat effect for subsequent analyses.

### Differential expression analysis

As described above in Section 2, we used the R/Bioconductor limma package and iterative Group Analysis (iGA) method for differentially expressed gene and GO term/pathway identification, respectively.

### COMBINER

**Figure 4:**
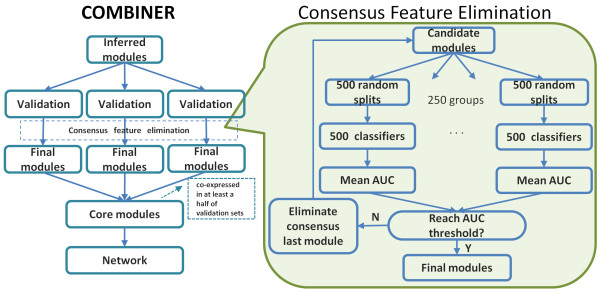
**Schematic overview of COMBINER.** COMBINER first infers candidate modules as activity vectors from each pathway in an inference dataset. It then validates these modules in validation datasets by regenerating activity vectors and performing supervised classification. Finally, the modules present in at least half of the validation sets are considered to be core modules. The resulting core module markers are then projected onto a known protein-protein interaction network. We generated 250 groups of 500 classifiers in parallel using LDA with recursive feature elimination. Both the classifier AUC and weight vectors were computed, and each feature was then ranked by its average normalized weight. The most consistently low-ranking feature was then removed recursively until the average AUC threshold was achieved. At this point, the remaining markers were considered to comprise the final modules.

As shown in Figure 4, the COMBINER method first infers the statistically discriminative modules from an inference dataset, then validates them in various validation sets using consensus feature elimination. If a validated final module is co-expressed in at least half of the validation sets, then it is defined as a core module. Finally, we project these core modules onto known PPI networks. To remove features, we generated 250 groups of 500 classifiers in parallel and applied Linear Discriminant Analysis (LDA) with recursive feature elimination [52] to each to compute AUCs as well as weight vectors. Each feature was then ranked by its average normalized weight. The most consistently low-ranking feature was then removed recursively until the average AUC threshold of 0.75 was achieved. At this point, the remaining features were considered to comprise the final modules.

In our previous work [28], we applied both the Condition Responsive Genes (CORG) [53] and Core Module Inference (CMI) [28] methods to infer candidate modules and express them as pathway activities (PAs). In the greedy search process, CORG picks up either up- or down-regulated genes, while CMI identifies genes of both directions together. However, because of the multiple-time-point nature of the social defeat data, the application of the CMI method is not straightforward. Thus, in this work we used only the CORG method with the procedure described as follows. For a given pathway, we first rank the standardized gene expressions by their limma moderated t-score. If up-regulated genes are dominant, we rank the t-score in descending order; otherwise, ascending order is chosen. Next, we aggregate the first two genes using the formula $y=\left(x_{1}+x_{2}\right)/\sqrt{2}$; if the expression of this aggregate yields a larger absolute t-score than the first gene, this combination is retained as a module with the combined expression becoming the PA. Otherwise, the procedure further adds the third gene using $y=\left(x_{1}+x_{2}+x_{3}\right)/\sqrt{3}$, and so on, until the module-size limit, 25 genes, is reached. Finally, we ranked all modules using the absolute value of the pathway activity t-score.

We faced two major challenges when modifying our COMBINER method. First, the multiple-time-point nature of the data initially decreased the binary classification performance of the static LDA classifier [52]. Second, the small data sample size leads to a large variability of feature ranks after recursive feature elimination. To cope with the first challenge, we used a linear model to deconvolve the time effects from the original expression values. We solved the second problem by improving our method for consensus feature elimination. We generated 250 groups of 500 classifiers in parallel, then removed the bottom feature using the voting principle. In general, using additional groups of classifiers will further improve the reproducibility of the final modules. In our experience 250 groups were sufficient to yield a reproducible result (results not shown). Finally, we used a fixed average AUC threshold to determine the final modules instead of the max average AUC threshold described in [28]. This was required since the inference and validation sets can be very dissimilar, which leads to low values of the max average AUC.

We obtained pathway information from the MSigDB v3.0 Canonical Pathways subset [54]. To decrease redundancy, we applied pathway filtering to remove bulky pathways. This resulted in a pathway dataset containing 791 pathways with 5,633 genes assayed in all regions. The protein-protein interaction information was obtained from String v9.0 [55].

### Average time curve

Let *y*_*ij*_ be the relative expression level of gene *i* at the sampling time *j*. The time-point expression patterns were modelled as follows,

(2) $$y_{\mathrm{ij}}=\mu_{i}\left(t_{j}\right)+\epsilon_{\mathrm{ij}}$$

where $\mu_{i}\left(t_{j}\right)=\log_{2}\left(\left|{\bar{x}}^{D}\left(t_{j}\right)/{\bar{x}}^{C}\left(t_{j}\right)\right|\right)$ is the population average time curve for gene *i* evaluated at time *t*_*j*_ and where *ϵ*_*ij*_ is the random deviation from this curve. ${\bar{x}}^{D}\left(t_{j}\right)$ and ${\bar{x}}^{C}\left(t_{j}\right)$ are average expressions of disease and control mice respectively for gene *i* at time *t*_*j.*_

### Software

COMBINER was implemented in Matlab R2010a with Bioinformatics toolbox v3.5 (Math Works Inc., Natick, MA), statconn (http://www.statconn.com/), LinkR (http://www.mathworks.com/matlabcentral/fileexchange/5051), and R [56]. The source code can be found in Additional files 6 and 7.

### Availability of supporting data

The supporting data is provided in the supporting information.

## Competing interests

The authors declare that they have no competing interests.

## Authors’ contributions

RY, BJD, SYM, RH, MJ, LP, and FJD conceived and designed the research. BJD performed the overlap analysis, RY developed the COMBINER computations. SYM, RH, and MJ provided the experimental data. RY and BJD wrote the paper. All authors read and approved the final manuscript.

## Supplementary Material

Additional file 1: Table S1 Significantly overlapping DEPATHs between Blood and Hemibrain. Click here for file

Additional file 2: Table S2 Significantly overlapping DEGOs between Blood and Septal Region. Click here for file

Additional file 3: Table S3 List of blood-brain core modules with associated genes. Click here for file

Additional file 4: Table S5 Biological Significance of blood core gene markers from literature mining. Click here for file

Additional file 5: Table S4 List of brain-brain core modules with associated genes. Click here for file

Additional file 6 **Processed microarray data used in the paper.** Click here for file

Additional file 7 Source code. Click here for file

## Disclaimers

The views, opinions, and/or findings contained in this report are those of the authors and should not be construed as official Department of the Army position, policy, or decision, unless so designated by other official documentation. Research was conducted in compliance with the Animal Welfare Act, and other Federal statutes and regulations relating to animals and experiments involving animals and adheres to principles stated in the Guide for the Care and Use of Laboratory Animals (NRC 2011) in facilities that are fully accredited by the Association for the Assessment and Accreditation of Laboratory Animal Care, International. Citations of commercial organizations or trade names in this report do not constitute an official Department of the Army endorsement or approval of the products or services of these organizations.
