# Supplementary material for: Core modular blood and brain biomarkers in social defeat mouse model for post traumatic stress disorder
Source: BMC Syst Biol. 2013 Aug 20;7:80. doi: 10.1186/1752-0509-7-80 (PMC3751782; doi:10.1186/1752-0509-7-80)
Supplement: Additional file 1: Table S1 — Significantly overlapping DEPATHs between Blood and Hemibrain. [file 1752-0509-7-80-S1.docx]

| **PATH ID** | **Name** | **Number of significant genes** | **p-value** | **FDR** |
| --- | --- | --- | --- | --- |
| 289 | KEGG RIBOSOME | 10 | <0.001 | <0.098 |
| 496 | REACTOME FORMATION OF A POOL OF FREE 40S SUBUNITS | 10 | <0.001 | <0.098 |
| 738 | REACTOME TRANSLATION | 10 | <0.001 | <0.098 |
| 752 | REACTOME VIRAL MRNA TRANSLATION | 10 | <0.001 | <0.098 |
| 537 | REACTOME GTP HYDROLYSIS AND JOINING OF THE 60S RIBOSOMAL SUBUNIT | 10 | 0.001 | 0.098 |
| 621 | REACTOME PEPTIDE CHAIN ELONGATION | 10 | 0.001 | 0.098 |
| 817 | REACTOME RNA POLYMERASE I III AND MITOCHONDRIAL TRANSCRIPTION | 10 | 0.001 | 0.098 |
| 653 | REACTOME REGULATION OF BETA CELL DEVELOPMENT | 10 | 0.002 | 0.104 |
| 654 | REACTOME REGULATION OF GENE EXPRESSION IN BETA CELLS | 10 | 0.002 | 0.104 |
| 723 | REACTOME TELOMERE MAINTENANCE | 10 | 0.002 | 0.104 |
| 734 | REACTOME TRANSCRIPTION | 10 | 0.002 | 0.104 |
| 551 | REACTOME INFLUENZA LIFE CYCLE | 10 | 0.003 | 0.132 |
| 552 | REACTOME INFLUENZA VIRAL RNA TRANSCRIPTION AND REPLICATION | 10 | 0.003 | 0.132 |
| 556 | REACTOME INSULIN SYNTHESIS AND SECRETION | 10 | 0.005 | 0.191 |
| 802 | REACTOME RNA POLYMERASE I PROMOTER OPENING | 10 | 0.007 | 0.237 |
| 795 | REACTOME VITAMIN B5 (PANTOTHENATE) METABOLISM | 8 | 0.009 | 0.283 |
| 396 | KEGG SYSTEMIC LUPUS ERYTHEMATOSUS | 10 | 0.010 | 0.284 |
| 576 | REACTOME METABOLISM OF PROTEINS | 10 | 0.013 | 0.318 |
| 470 | REACTOME DIABETES PATHWAYS | 10 | 0.016 | 0.381 |
| 670 | REACTOME RNA POLYMERASE I PROMOTER CLEARANCE | 9 | 0.021 | 0.440 |
| 641 | REACTOME PREFOLDIN MEDIATED TRANSFER OF SUBSTRATE TO CCT TRIC | 6 | 0.036 | 0.547 |
| 389 | KEGG BLADDER CANCER | 9 | 0.041 | 0.547 |
| 17 | BIOCARTA CHEMICAL PATHWAY | 4 | 0.042 | 0.552 |
| 295 | KEGG PROTEASOME | 10 | 0.046 | 0.570 |

**Table S1: Significantly overlapping DEPATHs between Blood and Hemibrain.** We list sub-pathways as before with information deriving from the analysis conducted on the Blood data. We note the prevalence of insulin-/diabetes-related sub-pathways in this list, which suggests similarity to the KEGG TYPE I DIABETES MELLITUS sub-pathway present in the overlap between Amygdala and Hippocampus (Table 1).
