# Supplementary material for: Core modular blood and brain biomarkers in social defeat mouse model for post traumatic stress disorder
Source: BMC Syst Biol. 2013 Aug 20;7:80. doi: 10.1186/1752-0509-7-80 (PMC3751782; doi:10.1186/1752-0509-7-80)
Supplement: Additional file 2: Table S2 — Significantly overlapping DEGOs between Blood and Septal Region. [file 1752-0509-7-80-S2.docx]

| **GO ID** | **Name** | **Number of significant genes** | **p-value** | **FDR** |
| --- | --- | --- | --- | --- |
| 1535 | POSITIVE REGULATION OF CALCIUM ION TRANSPORT INTO CYTOSOL | 2 | 0.001 | 0.134 |
| 4533 | GLYCOSYLATION | 16 | 0.002 | 0.200 |
| 791 | PROTEIN AMINO ACID GLYCOSYLATION | 16 | 0.003 | 0.261 |
| 3147 | MACROMOLECULE GLYCOSYLATION | 16 | 0.003 | 0.261 |
| 1460 | DETECTION OF CHEMICAL STIMULUS | 7 | 0.005 | 0.362 |
| 2175 | APOPTOTIC NUCLEAR CHANGES | 5 | 0.008 | 0.453 |
| 4379 | HEART GROWTH | 1 | 0.010 | 0.475 |
| 922 | ATP BIOSYNTHETIC PROCESS | 57 | 0.011 | 0.475 |
| 4146 | DETECTION OF STIMULUS | 70 | 0.011 | 0.475 |
| 3209 | RESPONSE TO ESTROGEN STIMULUS | 1 | 0.014 | 0.503 |
| 156 | T CELL HOMEOSTATIC PROLIFERATION | 2 | 0.016 | 0.503 |
| 3480 | REGULATION OF FEMALE RECEPTIVITY | 1 | 0.016 | 0.503 |
| 190 | RELEASE OF CYTOCHROME C FROM MITOCHONDRIA | 4 | 0.017 | 0.503 |
| 1011 | NUCLEUS ORGANIZATION | 6 | 0.018 | 0.503 |
| 1442 | RESPONSE TO HEAT | 2 | 0.018 | 0.503 |
| 4328 | FEMALE MATING BEHAVIOR | 1 | 0.018 | 0.503 |
| 4235 | CARDIAC MUSCLE TISSUE GROWTH | 1 | 0.019 | 0.503 |
| 3508 | ATP METABOLIC PROCESS | 59 | 0.020 | 0.503 |
| 4274 | CARDIAC MUSCLE CELL PROLIFERATION | 1 | 0.020 | 0.503 |
| 2365 | INDUCTION OF PROGRAMMED CELL DEATH IN RESPONSE TO CHEMICAL STIMULUS | 3 | 0.027 | 0.575 |
| 2366 | INDUCTION OF APOPTOSIS IN RESPONSE TO CHEMICAL STIMULUS | 3 | 0.028 | 0.583 |
| 2178 | BONE MINERALIZATION | 2 | 0.030 | 0.583 |
| 2625 | MUSCLE CELL PROLIFERATION | 1 | 0.031 | 0.590 |
| 3896 | POSITIVE REGULATION OF EPITHELIAL CELL PROLIFERATION | 1 | 0.044 | 0.683 |
| 1398 | PURINE RIBONUCLEOSIDE TRIPHOSPHATE BIOSYNTHETIC PROCESS | 63 | 0.046 | 0.698 |
| 1737 | ENERGY COUPLED PROTON TRANSPORT, DOWN ELECTROCHEMICAL GRADIENT | 17 | 0.049 | 0.715 |

**Table S2: Significantly overlapping DEGOs between Blood and Septal Region.** We list Biological Process GO terms with information deriving from the iGA GO term analysis conducted on the Blood data. We note the prevalence of apoptosis-related GO terms in this list, which suggests similarity to the SA CASPASE CASCADE sub-pathway present in the overlap between Hippocampus and Striatus Terminalis (Table 2).
